# Surface Modification of Electroosmotic Silicon Microchannel Using Thermal Dry Oxidation

**DOI:** 10.3390/mi9050222

**Published:** 2018-05-07

**Authors:** Tuan Norjihan Tuan Yaakub, Jumril Yunas, Rhonira Latif, Azrul Azlan Hamzah, Mohd Farhanulhakim Mohd Razip Wee, Burhanuddin Yeop Majlis

**Affiliations:** 1 Institute of Microengineering and Nanoelectronics (IMEN), Universiti Kebangsaan Malaysia (UKM), 43600 UKM Bangi, Selangor, Malaysia; tuan_norjihan@siswa.ukm.edu.my (T.N.T.Y.); rhonira@ukm.edu.my (R.L.), azlanhamzah@ukm.edu.my (A.A.H.); m.farhanulhakim@ukm.edu.my (M.F.M.R.W.); burhan@ukm.edu.my (B.Y.M.); 2 Department of Electronics Engineering, Faculty of Engineering, Universiti Teknologi MARA, 40450 Shah Alam, Selangor, Malaysia

**Keywords:** surface modification, electroosmotic flow, microfluidic, silicon nanochannel, thermal oxidation

## Abstract

A simple fabrication method for the surface modification of an electroosmotic silicon microchannel using thermal dry oxidation is presented. The surface modification is done by coating the silicon surface with a silicon dioxide (SiO_2_) layer using a thermal oxidation process. The process aims not only to improve the surface quality of the channel to be suitable for electroosmotic fluid transport but also to reduce the channel width using a simple technique. Initially, the parallel microchannel array with dimensions of 0.5 mm length and a width ranging from 1.8 µm to 2 µm are created using plasma etching on the 2 cm × 2 cm silicon substrate <100>. The oxidation of the silicon channel in a thermal chamber is then conducted to create the SiO_2_ layer. The layer properties and the quality of the surface are analyzed using scanning electron microscopy (SEM) and a surface profiler, respectively. The results show that the maximum oxidation growth rate occurs in the first 4 h of oxidation time and the rate decreases over time as the oxide layer becomes thicker. It is also found that the surface roughness is reduced with the increase of the process temperature and the oxide thickness. The scallop effect on the vertical wall due to the plasma etching process also improved with the presence of the oxide layer. After oxidation, the channel width is reduced by ~40%. The demonstrated method is suggested for the fabrication of a uniform channel cross section with high aspect ratio in sub-micro and nanometer scale that will be useful for the electroosmotic (EO) ion manipulation of the biomedical fluid sample.

## 1. Introduction

The past decade has seen the rapid advancement of microfluidic chip development. The main reason is because of the fluid flow characteristics in the micrometer structure that allows for an extremely slow fluid movement, less sample volume consumption and precision in fluid control. In microfluidic systems, the electroosmotic device (EO) becomes one of the most important parts in which the fluid can be manipulated by the electric potential between inlet and outlet. EO flow mechanism in a microchannel has been used for numerous microfluidic applications, including for fluids transport and manipulation [1], charge separation and ion transport [2] and fluids pumps [3]. As an example, the EO flow mechanism has been applied to transport fluids as in electroosmotic pump (EOP) [4]. The system was capable of generating a maximum flow rate of 15 µL/min, a significant amount of flow rate per device volume for a microfluidic system. The microchannel width in the fabricated pump is much greater than the height, to ease the fabrication process. Such designs suffer from clogging due to structure collapsing after bonding process for microsystem integration. Therefore, a microchannel structure for EO device requires a sufficient high channel aspect ratio (H/W >> 1) to create a uniform electric field distribution along the channel that induces a stable electroosmotic flow. For ion separation purposes, a small channel cross section is necessary due to stronger ion interaction between the charged system (ionic solution and charged channel surface) [5].

Microfluidic channels are typically fabricated in glass, silicon and polymeric substrate. The fabrication procedures of polymeric material like Polydimethylsiloxane (PDMS) and Poly (methyl methacrylate) (PMMA) offer a significantly lower cost and ease fabrication procedures [6,7]. Despite this, the polymeric based materials have a lower wall zeta potential and heat dissipation rates as compared to glass because glass has superior chemical properties for surface electrostatics reactions. Furthermore, a few methods to modify the PDMS and PMMA based microchannel surface have been previously reported. For instance, the plasma-based technique to tune the wettability of the surface has been demonstrated [8,9], indicating that a surface modification is preferable for improving the quality of the channel surface.

Glass also has an excellent dielectric property that is important for electroosmotic flow [10]. However, the glass fabricating technique is a major drawback due to its low etching rate, which must be considered when designing a glass based EO microfluidic device.

On the other hand, silicon material has been, for many years, established as the material to employ for a wide range of microelectronics devices and applications. A silicon microchannel has similar electrical properties to a glass substrate and it can be fabricated using the well-established integrated circuit (IC) fabrication technology [11]. Reactive ion etching using SF_6_ plasma is widely used to create uniform micro-channels with a vertical wall for microfluidic devices but the wall quality is poor due to the scallop effect, resulting from the repetitive alternating phase of passivation gas (C_4_F_8_) deposition and the etching gas (SF_6_) on the targeted wall. Some work on reducing the scallop effects by optimizing the deep reactive ion etching (DRIE) parameter has been reported. However, this complicated studies, due to the additional gas composition [12], an optimum etch and passivation cycle time [13] as well as the controlled flow rates of the etching/passivation ratio [14].

In this paper, we report the implementation of the dry oxidation process to grow an oxide layer on a prefabricated silicon based microfluidic channel in order to improve its surface quality [15] and at the same time to increase the channel aspect ratio by reducing the channel dimension down to the nanoscale for ion transport purposes. This method is also preferable because it is simple, compatible with the complementary metal-oxide-semiconductor (CMOS) process and eliminates the complex nanolithography process.

## 2. Microchannel Electroosmotic System Design

The principle of electroosmotic ion transport is based on the spontaneous reaction of a solid surface in contact with an ionic solution. For a negative surface charge, an accumulation of positive charge forms a stern layer in an electrical double layer (EDL) on the capillary wall. Therefore, the EO flow mechanism is produced by way of the induction principle of cations in the diffuse layer in the electric double layer (EDL), which move with the presence of the electric field. The cations migration will drag the bulk fluid in the capillary towards the negative potential in the flat flow profile.

**Figure 1:**
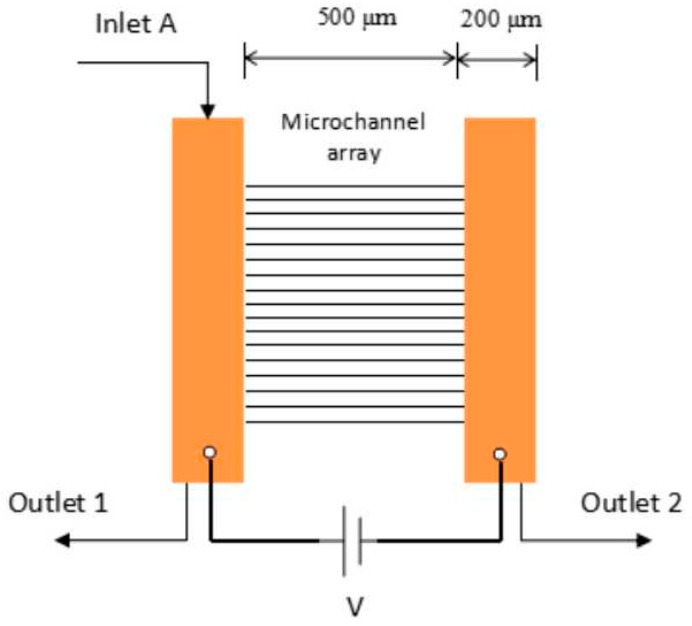
The microchannel electroosmotic system.

The microfluidic EO system consists of several surface–modified microfluidic channels in parallel array with an SiO_2_ coating with a length of 500 µm that is fabricated on a 390 µm thick silicon wafer <100>. The channel shape is rectangular with a width smaller than 1 µm and a depth of about 2 µm. A microchannel input (Inlet A—Outlet 1), having a channel geometry of 200 µm width and 20 mm length are integrated with the SiO_2_ coated silicon microchannel array. On the other end of the surface modified channel, the outlet channel (outlet 2 with similar dimensions to the inlet channel is integrated. The inlet port contains buffer and ionic solution that will be transported through the microchannel array towards the outlet reservoir by electroosmotic flow. For that purpose, a voltage potential V is applied across the microchannel array to create an electric field as shown in Figure 1.

In this work, the SiO_2_ coated silicon microchannel reduces not only the channel cross-section dimension but also improves the surface quality. To create micron size rectangular microchannels, we utilized standard photolithography and the reactive ion etching technique. The thermal oxidation was performed under atmospheric pressure with a constant O_2_ stream flow of 2000 mL/min at various process temperatures. Through the conducted procedures, a uniform oxide growth on both vertical and horizontal surfaces and SiO_2_ rectangular channels of a uniform size and shape in parallel array are produced.

**Figure 2:**
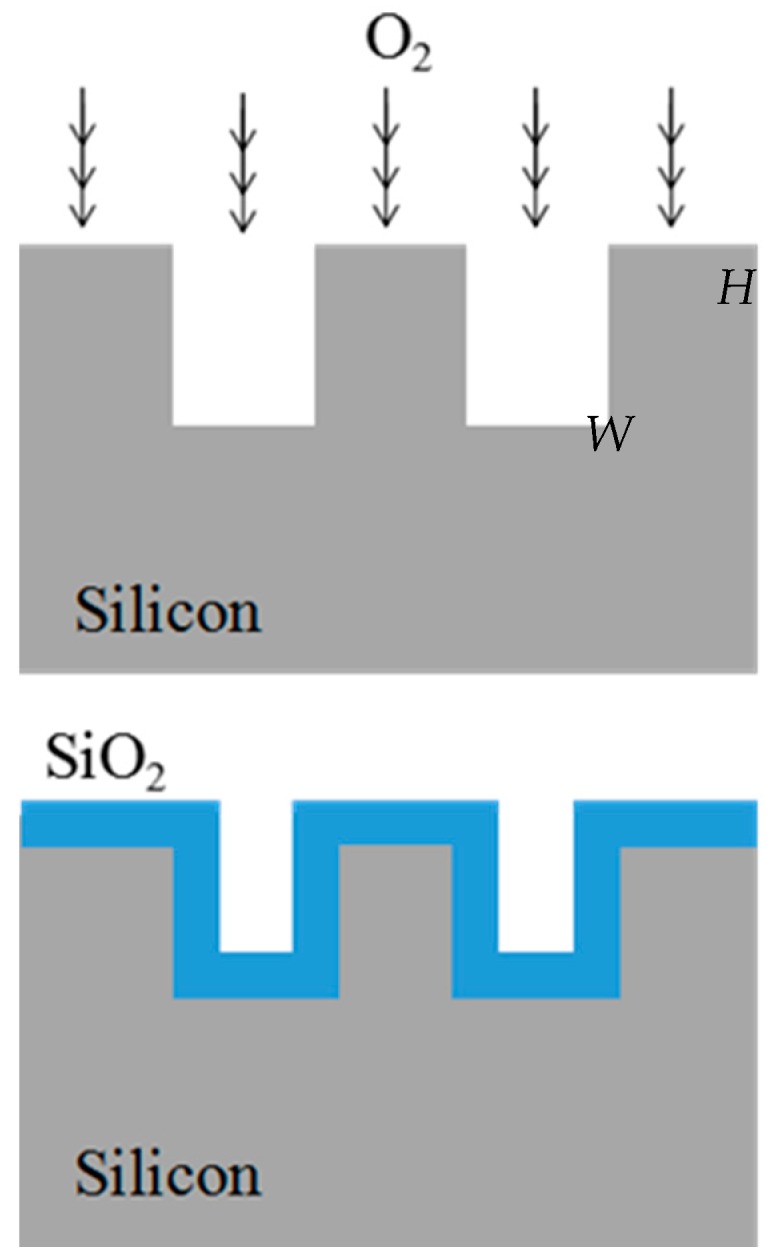
Mechanism of dry oxidation on silicon microchannel.

As shown in Figure 2, the oxidation process should produce an approximately uniform oxide growth rate on both the horizontal and vertical silicon walls. Hence, accurate control of the depth to width ratio (H/W) of the microchannels can be achieved after the oxidation process. The final dimension width of the electroosmotic channel is in the range below 1 µm with a higher aspect ratio.

## 3. Fabrication Process of Electroosmotic Device (EO) Channel System

### 3.1. Fabrication of Silicon Microchannel Using Plasma Etching Procedure

**Figure 3:**
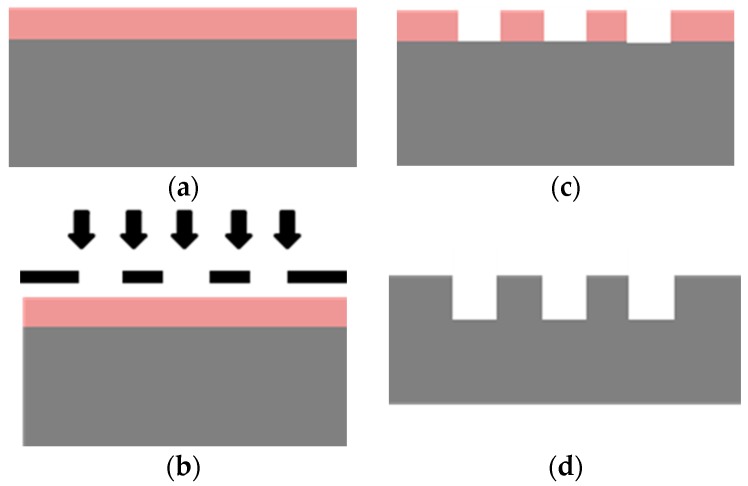
The diagram of silicon microchannel fabrication process (**a**) Photoresist as etching mask coating; (**b**) Transfer pattern using photolithography; (**c**) Pattern development using resist developer; (**d**) Si DRIE along the unprotected microchannel lines & photoresist removal.

Figure 3 shows the process flow to create the Si-microchannel. Initially, a set of electroosmotic microchannel arrays was fabricated using the standard photolithography and deep reactive ion etching (DRIE) on the silicon wafer <100>. The parallel microchannels were designed to have a length of 0.5 mm and a variation of widths from 1.8 µm to 2 µm. The microchannel was formed in a straight line and the distance between each channel was set to 5 µm.

The microchannel design was transferred onto a cleaned silicon surface by exposing the substrate coated with a positive photoresist under UV-light exposure. To pattern a vertical microchannel on the silicon substrate, we employed the high-density reactive ion SF_6_ etching, with passivation gas C_4_H_8_ and O_2_ at cryogenic temperature with an approximate etch rate of 1 µm per minute. After the DRIE process, the mask resist was removed and the geometries of the microchannels array were observed using a scanning electron microscope (SEM).

### 3.2. Surface Modification of Fabricated Silicon Microchannel Using Thermal Oxidation

The surface modification of the prefabricated silicon microchannel was done using an oxidation process in a high thermal ambience. Basically, the oxide was grown on the heated silicon surface at a very high temperature between 800–1200 °C with the presence of oxygen gas flow or water vapor, referring to dry and wet oxidation respectively. In this work, we created the SiO_2_ microchannel based on the oxidation of silicon in dry flowing oxygen at atmospheric pressure in three different process temperatures, 990 °C, 1020 °C and 1040 °C. The formation of silicon dioxide layer on silicon channels was described by the chemical reaction below:

Si + O_2_ = SiO_2_ (1)

In dry thermal oxidation, O_2_ molecules diffuse through the surface oxide layer and react with the silicon atom at the Si-SiO_2_ interface. The resulting SiO_2_ surface layer is not coplanar with the original silicon surface, which means 0.44*d* of silicon is consumed for a thickness of *d* oxide layer growth in the thermal dry oxidation process.

Before starting the oxidation procedure, the etched sample was cleaned in acetone and a methanol ultrasonic bath for five minutes each followed by rinsing the substrate in DI water for 1 min. The sample was then soaked in 10% HF solution for 60 s to eliminate the undesired native oxide layer before it was rinsed again in DI water and dried with nitrogen.

**Figure 4:**
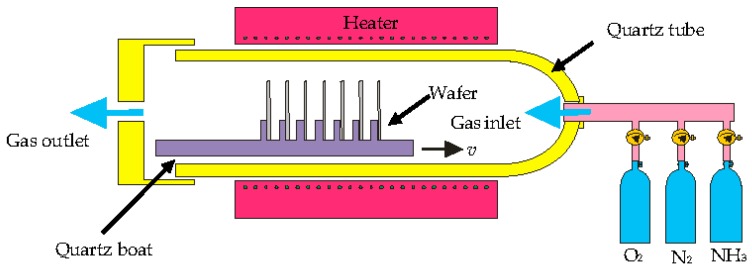
Schematic diagram of oxidation furnace.

Then, the samples were placed in the ceramic boat and were put at the mouth of the oxidation glass tube furnace that had previously been filled up with nitrogen (N_2_) gas at a 2000 mL/min stream rate. The resistance heater on the tube furnace was then heated up according to the targeted process temperature. After the furnace temperature reached the required temperature, the sample boat was pushed to the heated zone, which is at the center of the furnace tube. The oxidation process began after we stopped the supplied N_2_ gas and started to flow the dried oxygen gas into the tube furnace under a constant flow rate of 2000 mL/min. The oxidation process was maintained under a constant oxygen flow rate at atmospheric pressure from 4 to 12 h. Figure 4 shows the oxidation furnace apparatus for thermal dry oxidation. A uniform color scheme with no surface abnormality appeared on the oxidized sample’s surface at all experiment conditions. The morphology of SiO_2_ growth surface on the sample was scanned using F50 Thin Film Metrics at 20 different points while the cross-section of the SiO_2_ microchannel was verified using the scanning electron microscope.

## 4. Results and Discussion

### 4.1. SiO_2_ Layer Thickness and Oxidation Growth Rate

**Figure 5:**
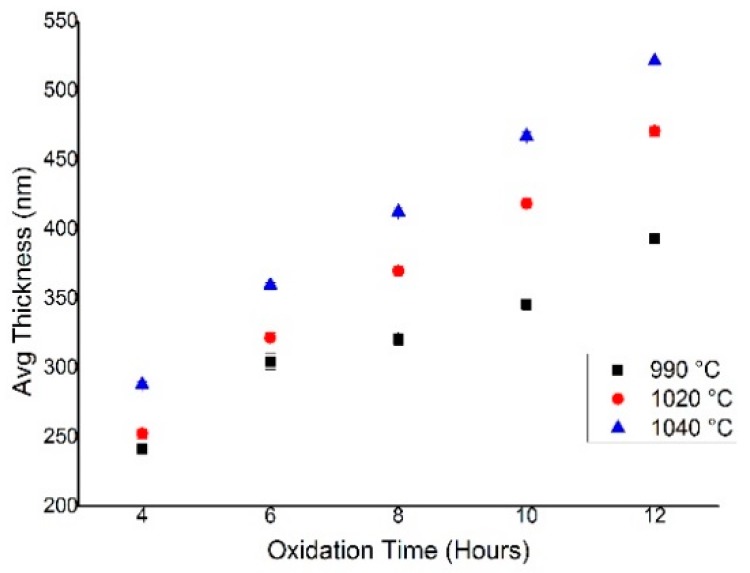
Oxide thickness against oxidation time of thermal dry oxidation for silicon <100>.

The average thickness of the oxide layer on the silicon microchannel was highly controlled by the process temperature and the oxidation time. A plot of average oxide thickness against time at various process temperatures is shown in Figure 5. In the time range of 4 to 12 h for all oxidation temperatures, the oxidation layer thickness was found to increase with the oxidation time. In addition, the higher the process temperature, the thicker the oxide layer will be grown. This relationship of temperature and time for the oxide thickness was well agreed with the finding reported in Reference [16]. From the plot, we perceived the maximum thickness of 520 nm oxide grown at 1040 °C for 12 h of oxidation time.

**Figure 6:**
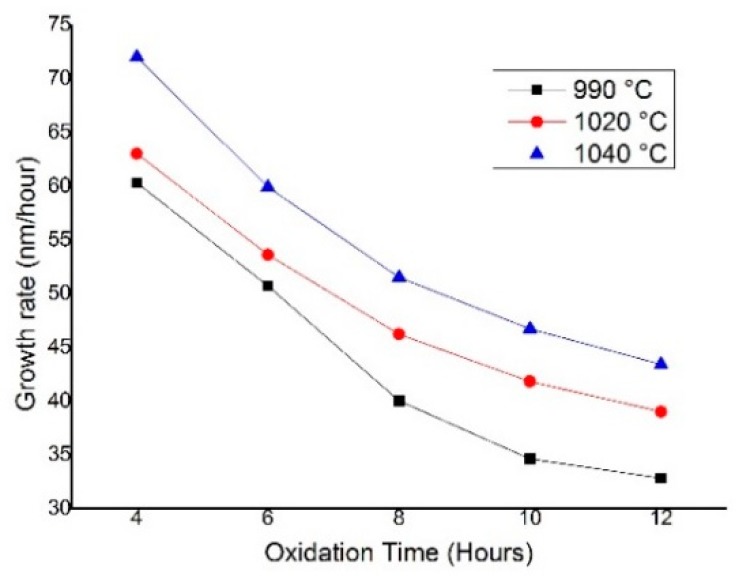
Oxide growth rate of thermal dry oxidation for silicon <100>.

The oxide thickness was measured at intervals after 4, 6, 8, 10 and 12 h of the oxidation process. The highest growth rate was observed for the first 4 h of oxidation time for all process temperatures. The growth rate at all conducted process temperatures is presented in Figure 6. It is shown that for the first 4 h of oxidation, the maximum growth rate was up to 72 nm/h at 1040 °C. As the oxidation time increased, we observed a consistent decrease of growth rate. The same trend was observed for other process temperatures of 1020 °C and 990 °C. The decreasing growth rate by time can be explained due to a lower supply of oxygen atoms at the silicon interface as the thickness of the oxide layer increases. We also found that the maximum oxide growth rate was 33% higher with an increase of 50 °C in the process temperature for 12 h of oxidation time. This finding proved the strong relation between the temperature and the oxide growth rate as the oxidant diffusivity increased with the increasing temperature [11,16,17].

### 4.2. Surface Uniformity

The thickness of the oxide layer on the sample surface at all oxidation durations was found to be uniform across a 2 cm × 2 cm sample area. The thickness measurements were characterized using F50 Filmetrics at 20 random scanned points across the samples. The measurement principle was based on the light scattering effect of the incident light normal to the thin film surface.

**Figure 7:**
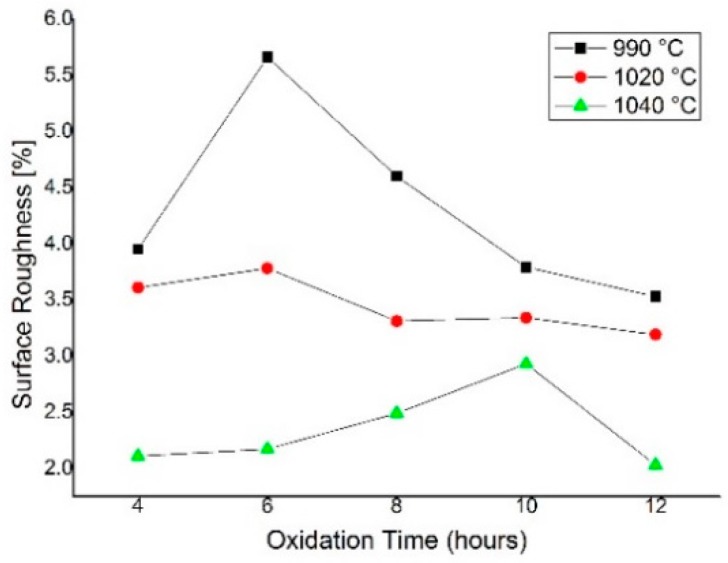
Surface roughness of oxide layer for various process temperature.

Figure 7 shows the surface roughness, indicating the uniformity of the oxide layer thickness that is calculated as the difference between the maximum and minimum thickness of the grown oxide layer divided by the average thickness at every oxidation temperature. In general, the oxide thickness measurement showed that the oxide layer was grown with the lowest roughness as the oxidation temperature increased. The surface roughness of 2.03% was observed for the oxidation temperature of 1040 °C. The oxide surface roughness also improved when we prolong the oxidation time to 12 h. The longer oxidation time allows for a thicker oxide layer growth with better uniformity of the microchannel surface.

**Figure 8:**
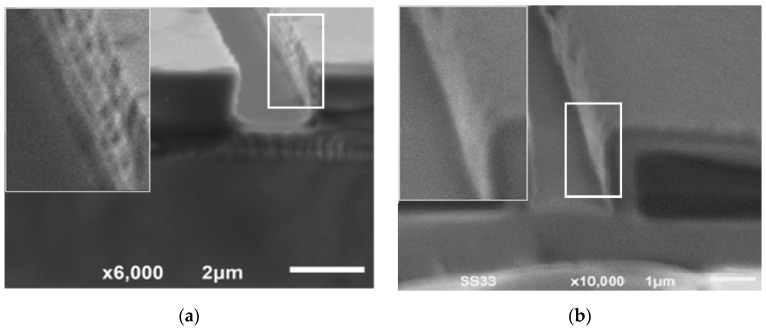
Scallop effect on the silicon vertical wall (**a**) before and (**b**) after oxidation.

Figure 8 shows the SEM observations on the EO channel before and after oxidation process. The scallop effect becomes a major challenge for high aspect ratio microchannel fabrication using DRIE [18]. The scallop roughness, as shown in Figure 8a, was not desirable especially for an electroosmotic microfluidic system because it can create unnecessary flow characteristics and induce unwanted pressure inside the microchannel. On top of that, the surface roughness can change the EDL properties near the surface wall, hence reduce the electroosmotic flow inside the microchannel [19,20]. Figure 8b shows the effect of oxidation on the side wall smoothness. It can be seen that the scallop roughness on the vertical wall of the microchannel resulted from the plasma etching process was improved by the oxide layer growth on the silicon vertical wall after 12 h of oxidation.

### 4.3. Microchannel Structure Improvement after Thermal Oxidation

**Figure 9:**
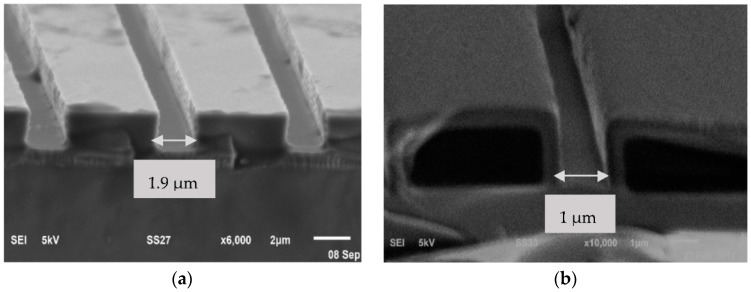
(**a**) Silicon microchannel before oxidation process; (**b**) SiO_2_ microchannel after 12 h thermal dry oxidation at 1040 °C.

A high aspect ratio SiO_2_ microchannel was successfully created by using dry thermal oxidation that is suitable for an electroosmotic flow application. The shape and cross section of the microchannel was uniform, as demonstrated by Figure 9a,b. The width of the microchannel was reduced to 42% with an oxide layer of 520 nm on the channel wall. The color scheme on the top surface of the substrate was seen consistently without any abnormal spots.

## 5. Conclusions

In conclusion, we presented a simple technique for fabricating a silicon electroosmotic (EO) channel with surface modification. The microchannel arrays were initially realized by reactive ion etching coupled with post processed thermal dry oxidation to produce high aspect ratio microchannels with an improved cross section and surface morphology. The strong relation between oxide thickness and the temperature was presented by performing the thermal dry oxidation at 990 °C, 1020 °C and 1040 °C. The oxide growth rate was found to increase at higher process temperatures. By using this method, the microchannel width was reduced by ~40% with the minimum aspect ratio (H/W) of 2. The surface quality was also improved as the scallop effect from the plasma etching process was reduced by adding an adequate amount of oxide layer. A uniform cross section with a good quality of surface roughness was essential for demonstrating a steady electroosmotic flow inside the microchannel. The final structure of the SiO_2_ microchannels array will be integrated with a PDMS structure to work as a complete electroosmotic microfluidic device. This process was foreseen to be able to produce a high aspect ratio sub-microchannel and nanochannels without implementing the nanolithography procedure that will be beneficiary for ion transport purposes.

## References

[1] Pennathur S., Santiago J.G. (2005). Electrokinetic transport in nanochannels. 2. Experiments. Anal. Chem..

[2] Buyong M.R., Larki F., Faiz M.S., Hamzah A.A., Yunas J., Majlis B.Y. (2015). A tapered aluminium microelectrode array for improvement of dielectrophoresis-based particle manipulation. Sensors.

[3] Zeng S., Chen C.H., Mikkelsen J.C., Santiago J.G. (2001). Fabrication and characterization of electroosmotic micropumps. Sens. Actuators B Chem..

[4] Chen C.H., Santiago J.G. (2002). A planar electroosmotic micropump. J. Microelectromech. Syst..

[5] Garcia A.L., Ista L.K., Petsev D.N., O’Brien M.J., Bisong P., Mammoli A.A., Brueck S.R.J., López G.P. (2005). Electrokinetic molecular separation in nanoscale fluidic channels. Lab Chip.

[6] Rodríguez-Ruiz I., Babenko V., Martínez-Rodríguez S., Gavira J.A. (2018). Protein separation under a microfluidic regime. Analyst.

[7] Masrie M., Majlis B.Y., Yunas J. (2014). Fabrication of multilayer-PDMS based microfluidic device for bio-particles concentration detection. Bio-Med. Mater. Eng..

[8] Vourdas N., Tserepi A., Boudouvis A.G., Gogolides E. (2008). Plasma processing for polymeric microfluidics fabrication and surface modification: Effect of super-hydrophobic walls on electroosmotic flow. Microelectron. Eng..

[9] Thorslund S., Nikolajeff S. (2007). Instant oxidation of closed microchannels. J. Micromech. Microeng..

[10] Kirby B.J., Hasselbrink E.F. (2004). Zeta potential of microfluidic substrates: 2. Data for polymers. Electrophoresis.

[11] Madou M.J. (2002). Fundamentals of Microfabrication.

[12] Abdolvand R., Ayazi F. (2008). An advanced reactive ion etching process for very high aspect-ratio sub-micron wide trenches in silicon. Sens. Actuators A Phys..

[13] Miller K., Li M., Walsh K.M., Fu X.A. (2013). The effects of DRIE operational parameters on vertically aligned micropillar arrays. J. Micromech. Microeng..

[14] Chang C., Wang Y.F., Kanamori Y., Shih J.J., Kawai Y., Lee C.K., Wu K.C., Esashi M. (2005). Etching submicrometer trenches by using the Bosch process and its application to the fabrication of antireflection structures. J. Micromech. Microeng..

[15] Barillaro G., Merlo S., Strambini L.M. (2008). Bandgap tuning of silicon micromachined 1-D photonic crystals by thermal oxidation. IEEE J. Sel. Top. Quantum Electron..

[16] Deal B.E., Grove A.S. (1965). General relationship for the thermal oxidation of silicon. J. Appl. Phys..

[17] Razeghi M. (2010). Technology of Quantum Devices.

[18] Gao F., Ylinen S., Kainlauri M., Kapulainen M. (2014). Smooth silicon sidewall etching for waveguide structures using a modified Bosch process. J. Micro/Nanolithogr. MEMS MOEMS.

[19] Qiao R. (2007). Effects of molecular level surface roughness on electroosmotic flow. Microfluid. Nanofluid..

[20] Masilamani K., Ganguly S., Feichtinger C., Bartuschat D., Rüde U. (2015). Effects of surface roughness and electrokinetic heterogeneity on electroosmotic flow in microchannel. Fluid Dyn. Res..

